# The Association Between Sociodemographic and Socioeconomic Factors and Meeting the Recommended Physical Activity Guidelines in Older Adults in the U.S.

**DOI:** 10.3390/ijerph23030344

**Published:** 2026-03-09

**Authors:** Betty R. Sierra Tamargo, Pura Rodríguez de la Vega, Noël C. Barengo

**Affiliations:** Department of Medical Education, Herbert Wertheim College of Medicine, Florida International University, Miami, FL 33199, USA; bsier014@fiu.edu (B.R.S.T.);

**Keywords:** physical activity guidelines, socioeconomic factors, sociodemographic factors, cross-sectional study, exercise

## Abstract

**Highlights:**

**Public health relevance—How does this work relate to a public health issue?**

- In older adults, physical inactivity is a public health issue because it increases the risk of older adults suffering from chronic diseases, falls, functional decline, and early mortality.
- This study highlights socioeconomic and sociodemographic determinants affecting older adults’ compliance with physical activity recommendations, highlighting how social and structural determinants of health influence their ability to engage in physical activity.

**Public health significance—Why is this work of significance to public health?**

- This work is significant to public health because understanding which groups may be less likely to meet physical activity recommendations enables the creation of targeted interventions and prevention strategies to reduce the burden of disability, disease, and healthcare use in an aging population.
- It is also significant because it fills in a critical gap in U.S.-based public health research by focusing on older adults, a population of interest given the scarce research focus they receive, as well as by examining the socioeconomic and sociodemographic factors that influence their adherence to physical activity recommendations, an area that has not been thoroughly explored.

**Public health implications—What are the key implications or messages for practitioners, policymakers, and/or researchers in public health?**

- Interventions must be tailored to target the unique barriers faced by older adults with lower income, lower educational level, advanced age, and chronic conditions, among others, who are more affected to ensure equitable access to feasible, safe, and culturally appropriate physical activity opportunities.
- Policy efforts must address social determinants, including affordability, neighborhood resources, and access to supportive environments. At the same time, researchers should continue exploring other strategies that could encourage participation among the most vulnerable older adult populations.

**Abstract:**

This study aimed to identify socioeconomic and demographic factors associated with meeting the recommended physical activity (PA) guidelines for older adults. This analytical, cross-sectional study used data from the 2022 NHIS, including 8189 older adults (≥65 years). Compliance with aerobic and strengthening PA guidelines was the main outcome variable. Covariates included sex, education, income, relationship status, race, housing stability, urban/rural county, health status, and region. Unadjusted and adjusted logistic regression analyses estimated odds ratios (ORs) and 95% confidence intervals (CIs). Only 14.4% (n = 1235) of older adults met the PA guidelines. Odds of complying decreased by 40% in NH single/multiple races (OR 0.6; 95% CI 0.4–1.0). Men had higher odds (OR 1.4; 95% CI 1.2–1.6) of adhering than women. Compared with those with a bachelor’s degree, the odds of adhering were 1.4 (95% CI 1.2–1.7) for graduate participants, 0.3 (95% CI 0.2–0.4) for participants with less than HS education, 0.4 (95% CI 0.3–0.5) for HS graduates, and 0.7 (95% CI 0.6–0.8) for those with some college education. The odds decreased by 40% for those with an income-to-poverty ratio threshold < 1 (OR 0.6; 95% CI 0.4–0.9), 1–1.99 (OR 0.6; 95% CI 0.4–0.7), and 2–3.99 (OR 0.6; 95% CI 0.5–0.8) compared with ≥5. Healthcare providers should develop targeted interventions to address individuals’ unique circumstances and reduce these inequities.

## 1. Introduction

Despite the many gains people could have from physical activity, most people in the United States remain insufficiently active; in other words, they are non-compliant with recommended physical activity guidelines, putting them at unnecessary risk of disease and specific conditions. Other studies have found that older age is one of the predictors of noncompliance with recommended PA guidelines in the U.S.; in other words, most older adults are sedentary [1,2]. In general, with increasing age, the probability of meeting recommended physical activity guidelines declines, indicating that older adults are the least likely to comply, making them the population of interest for this research [3,4,5,6,7,8,9].

The World Health Organization (WHO) provides evidence-based recommendations on the frequency, intensity, and duration of physical activity needed to support health across different age groups. For adults aged 65 years and older, the 2020 WHO guidelines emphasize the importance of multicomponent physical activity, which includes aerobic exercise, muscle-strengthening activities, and balance training. These activities should be adjusted to each individual’s functional ability and performed safely. The guidelines are supported by extensive research in older populations demonstrating strong relationships between physical activity, better physical function, and healthier aging. In particular, low activity levels are associated with higher fall risk and fall-related injuries, while multicomponent exercise programs that combine endurance, strength, balance, and functional training have been shown to improve stability and reduce fall-related harm [1,10,11,12,13].

Previous scientific research shows that there are differences in compliance with PA guidelines based on gender, educational levels, relationship status, race/ethnicity, and birthplace [2,3,4,5,6,7,8,9,10,11,12,13,14,15,16]. Despite the substantial body of literature on physical activity, there has been little research in the United States population, especially focusing specifically on the socioeconomic and sociodemographic factors that affect older adults’ ability to comply with physical activity recommendations. This is a gap in the literature that the present research aims to address.

The purpose of this study was to identify socioeconomic and sociodemographic factors associated with older adults’ ability to meet the recommended physical activity guidelines for aerobic and strengthening activity. It was hypothesized that different socioeconomic and sociodemographic statuses would affect older adults’ compliance with recommended physical activity guidelines.

## 2. Materials and Methods

### 2.1. Study Design and Population

This study employed an analytical cross-sectional design and conducted a secondary analysis of data from the 2022 National Health Interview Survey (NHIS). For this survey, the NHIS used a stratified, multistage sample design. Using a multistage probability sampling method, the survey randomly selected households, excluding certain groups like active-duty military and institutionalized individuals (those living in correctional facilities, nursing homes, and psychiatric hospitals), from all individuals living across the country in any of the fifty states or the District of Columbia. Since certain criteria had to be met for the interview, only fixed or stable households were selected. Therefore, this excluded people living in the streets who suffer from homelessness and those outside of the country. This method enables representative sampling of households and non-institutional group quarters. For the household selection, to streamline operations and reduce costs, the NHIS used geographically clustered sampling. This method divided the population into smaller clusters based on defined areas, such as counties or metropolitan regions, using more than 300 clusters of addresses within well-defined geographic areas. The NHIS conducted face-to-face interviews throughout 2022, with 27,651 adults participating; one adult aged 18 or older was randomly selected from each household in the sample, achieving a 47.7% response rate. The survey followed ethical guidelines and reporting standards for observational studies [2,17].

The main inclusion criteria for this study were age of 65 years or over and participation in the 2022 NHIS. The initial sample of participants after applying the inclusion criteria to the NHIS dataset was 8771. Participants with missing data on any NHIS items measuring the independent variables, the dependent variables, or the covariates relevant to this study were excluded from the analysis (n = 582). The final sample for the analysis was 8189. Table A1 presents the percentages of missing data for each variable.

### 2.2. Measurements/Variables

The main outcome variable was meeting the guidelines for aerobic and strengthening activity. Meeting the guidelines for aerobic activity was defined as doing at least 150 min (2 h and 30 min) to 300 min (5 h) of moderate-intensity aerobic physical activity per week, 75 min (1 h and 15 min) to 150 min (2 h and 30 min) of vigorous-intensity aerobic physical activity per week, or an equivalent combination. Strengthening activity was defined as activities of moderate or greater intensity that involve all major muscle groups on at least two days per week. Older adults, in particular, should engage in multicomponent physical activity that includes balance training, aerobic, and muscle-strengthening activities, adjusting their level of activity based on their capacity and safety to complete them, along with being as physically active as possible [1].

The main independent variables for this explorative study were socioeconomic and sociodemographic factors such as age (65–74 or 75–85), sex (male vs. female); educational level (less than high school, high school graduate, some college no degree/associate degree, bachelor’s degree (BA, AB, BS, BBA), or graduate degree (master’s or doctorate)); ratio of family income to poverty threshold (less than 1.00, 1.00–1.99, 2.00–3.99, 4.00–4.99, or 5.00 or greater); relationship status (married/living with a partner together as an unmarried couple or neither/no partner); race (Hispanic, NH White only, NH Black/African American only, or NH other single and multiple races); delayed access to healthcare based on insurance (yes or no); housing stability (less than a year, 1 to 3 years, 4 to 10 years, or more than 10 years); urban/rural classification of the county participants lived in (large central metro, large fringe metro, medium and small metro, or nonmetropolitan); household region (Northeast, Midwest, South, or West); and health factors, such as certain chronic conditions (COPD, emphysema, or chronic bronchitis; arthritis, rheumatoid arthritis, gout, lupus, or fibromyalgia; or dementia or Alzheimer’s disease), diabetes, cancer, and myocardial infarction (all dichotomized in yes or no). Table A2 presents the definitions of all variables used in this study.

### 2.3. Statistical Analysis

This study used descriptive analysis to analyze the frequency distribution of the qualitative variables. A bivariate analysis (chi-square) was employed to compare frequency distributions according to the main outcome variable. The covariates included in the model were identified based on previous scientific literature. At the analysis stage, bivariate analysis was conducted first to compare the frequency distributions of each covariate according to the main exposure and the main outcome variable to identify potential confounders. An alpha of <0.2 was used as a threshold to include a covariate into the logistic regression model. Variables that had a *p*-value above the set threshold were not included in the adjusted model. Collinearity diagnostics were used to assess the correlation between variables. Unadjusted and adjusted log regression analyses were used to calculate odds ratios and 95% confidence intervals. Analyses applied NHIS weights to generate nationally representative estimates and 95% CIs, accounting for the complex sample design and restricted to complete cases. Stata software version 19 was used for all statistical analyses.

### 2.4. Ethical Considerations

The study protocol was presented to the Internal Review Board (IRB) of the Florida International University Herbert Wertheim College of Medicine for approval. However, IRB approval was waived because this study was not considered a human-subjects study, as it used secondary, de-identified data.

## 3. Results

From a total of 8189 participants who were included in the study, 14.4% (n = 1235) met the recommended guidelines for both aerobic and strengthening physical activity, and 85.6% (n = 6954) did not.

Table 1 presents the distribution of sample characteristics according to whether adults met the recommended physical activity guidelines for aerobic and strength activities. The results showed that there was a statistically significant difference in the prevalence of adherence to the recommended physical activity guidelines according to sex, race, age, education level, the ratio of family income to poverty threshold, relationship status, urban/rural classification for the county, household region, having chronic respiratory conditions, having some form of arthritis, gout, lupus, or fibromyalgia, having dementia/Alzheimer’s, and having diabetes. Males (17.4%) complied more with the guidelines than females (11.9%; *p* < 0.001). Hispanic participants (9.6%) had lower compliance than NH White (15.7%), NH Black (10.5%), and other single and multiple races (10.8%), *p* < 0.001. In addition, participants aged 65–74 years (16.7%) had a 5.8% higher compliance rate than those aged 75–85 years (10.9%), *p* < 0.001. Moreover, participants who completed a graduate degree (30.3%) had a higher prevalence of adherence than those who completed a bachelor’s degree (22.9%), those with some college education or an associate degree (13.9%), high school graduates (7.3%), and those with less than high school education (4.3%), *p* < 0.001. In addition, while the prevalence of participants who complied with both guidelines with a ratio of family income to poverty threshold of 5.00 or greater was 24.2%, those with 4.00–4.99 had a prevalence of 17.3%, those with 2.00–3.99 had a prevalence of 10.7%, those with 1.00–1.99 had a prevalence of 7.5%, and those with less than 1.00 had a prevalence of 6.9%, *p* < 0.001. Participants who were married or living with a partner (16.4%) had a 5.1% higher prevalence of compliance than those without a partner (11.3%), *p* < 0.001. Additionally, participants who lived in nonmetropolitan areas (10.8%) complied less with the guidelines than those in medium and small metro (14.1%), large fringe metro (15.8%), and large central metro (15.7%) areas, *p* = 0.003. Those living in the West (17.5%) had higher levels of adherence than those in the South (12.5%), Midwest (14.8%), and Northeast (13.9%), *p* = 0.001.

Table 2 presents the adjusted logistic regression results for factors associated with meeting the recommended physical activity guidelines for aerobic and strengthening activities.

Age, gender, educational level, income, and household region were associated with meeting the physical activity recommendations in the adjusted model. Adjusted logistic regression analysis showed the odds of complying with the guidelines decreased by 30% in participants aged 75–85 (OR 0.70; 95% CI 0.61–0.82) and by 35% in NH single/multiple races (OR 0.65; 95% CI 0.43–0.98). Men had 1.4 times higher odds (OR 1.40; 95% CI 1.21–1.62) of meeting guidelines than women. Compared with those with a bachelor’s degree, the odds of adhering to the guidelines were 1.42 (95% CI 1.15–1.74) for graduate participants, 0.28 (95% CI 0.18–0.42) for those with less than a high school education, 0.38 (95% CI 0.30–0.48) for those who graduated from high school, and 0.68 (95% CI 0.55–0.82) for those with some college education. The odds decreased by 40% for those with an income-to-poverty ratio threshold of <1 (OR 0.60; 95% CI 0.42–0.86), 1–1.99 (OR 0.55; 95% CI 0.43–0.71), and 2–3.99 (OR 0.63; 95% CI 0.53–0.76) compared with a ratio of 5 or greater. Living in the West raised odds by 33% (OR 1.33; 95% CI 1.09–1.63) compared with South residents.

No statistically significant associations between complying with both guidelines were observed regarding cancer status, duration of residence, and delayed medical care due to lack of insurance in the unadjusted model. Therefore, these variables were not included in the final adjusted exploratory analysis.

## 4. Discussion

### 4.1. Overall Results

Extensive prior research has consistently shown that socioeconomic and sociodemographic factors—including sex, age, education, income, and race—are strongly associated with physical activity patterns in adults. Studies across diverse populations have demonstrated that physical inactivity is more common among older individuals, women, those with lower educational attainment, and those with lower income levels [1,2,3,4,5,6,7,8,9,14,16,18,19,20].

This study’s findings highlighted that differences in adherence to recommended physical activity guidelines among adults aged 65 years and older were associated with socioeconomic and sociodemographic factors, as well as certain health conditions. Among the factors that were shown to be associated with compliance with both aerobic and strengthening physical activity guidelines were sex, age, race, educational level, ratio of family income to poverty threshold, household region, having chronic respiratory conditions (COPD, emphysema, or chronic bronchitis), having some form of arthritis, gout, lupus, or fibromyalgia, having dementia or Alzheimer’s, and having diabetes. These findings align with the well-established existing body of research.

### 4.2. Main Findings

Male participants were more likely to comply with the guidelines than their female counterparts. Previous research supports these results, which have shown that women tend to have lower adherence to physical activity guidelines [8,9,10,14]. Similarly, in Scheers’ study, males were more likely than females to comply with guidelines [12]. Sun’s study also found that women were less likely to be regularly active [13]. A possible explanation for this finding, suggested by prior research, is that men appear to engage in physical activity for enjoyment. In contrast, women are more likely to engage in it to improve their health or appearance, so those who do not perceive a need to exercise may be less likely to meet recommended physical activity levels [18]. In this study, it was found that participants who belonged to NH single and multiple races were less likely to meet the recommended guidelines than NH White, NH Black, and Hispanic participants, which is supported by previous research that found Asian men to be less likely to engage in sufficient physical activity compared to White men [19]. Another study found lower non-work physical activity among other races—not including Black and Hispanic individuals—compared with NH Whites, and lower work physical activity among Asians relative to NH Whites [20]. This might be explained, as previous research suggests, by the fact that minority individuals typically reside in areas with lower access to physical-activity-promoting resources compared to their White counterparts, which may explain these differences in adherence to guidelines [19]. This study found that more participants aged 65–74 years complied with both physical activity guidelines than those aged 75–85 years. This is supported by prior research findings indicating that older age is associated with lower compliance [2,7,8,9,10,11,12,13]. A possible explanation for this is that as people age, they often experience a decline in physical health, including reduced muscle strength, joint issues, and chronic conditions that may make physical activity more challenging. Additionally, older adults may experience greater mobility limitations and a higher risk of falls, which can discourage them from engaging in physical activity. Social factors, such as reduced social support and fewer opportunities for physical activity, could also play a role.

Moreover, participants who completed a graduate degree were more likely to comply with the guidelines for both types of physical activity than those with a bachelor’s degree, who were more likely to comply than those with a lower educational level. These results were consistent with previous research, which has also found that lower educational levels are associated with lower compliance with physical activity guidelines [2,7,8,9,10,11,12,15]. Prior research has found that physical inactivity decreases as education increases; that adults with no qualifications are about three times more likely to be inactive than those with a degree; and that this education gradient remains strong even after accounting for age, gender, health status, occupation, and local area characteristics [21]. A hypothesized explanation is that individuals with higher education may have greater awareness of the health benefits of regular exercise, leading to more informed lifestyle choices. Additionally, higher educational attainment is often associated with higher socioeconomic status, which could provide greater access to resources such as gyms, recreational facilities, and safe environments for physical activity. People with higher levels of education may also have more flexible work schedules and better work–life balance, which could give them more time to engage in physical activity. Furthermore, social networks and peer influences within higher-educated groups often prioritize health and fitness, which could encourage individuals to maintain an active lifestyle. Previous research has also suggested that older adults with inadequate health literacy are significantly less likely to report regular physical activity [22]. It could be hypothesized that those with lower educational attainment have lower health literacy, thereby making it more difficult to make informed health-related decisions, such as engaging in physical activity to improve their health.

Furthermore, individuals farther from the poverty threshold exhibited higher compliance with physical activity guidelines, consistent with previous studies showing that higher-income participants engage more in physical activity. Other studies have also linked individual poverty to lower activity levels [2,15]. Previous studies have also found that participants with lower incomes exhibit lower adherence to physical activity guidelines [7,9]. A possible explanation could be that lower-income neighborhoods often have fewer resources and infrastructure to support physical activity. Additionally, individuals with lower incomes may face time constraints due to longer working hours, multiple jobs, and family responsibilities, leaving them with less time for exercise. Financial barriers, such as the cost of gym memberships, sports equipment, and fitness classes, can also limit opportunities for physical activity. Furthermore, lower-income individuals may have less access to health education and awareness about the benefits of physical activity.

This study showed that participants who lived in the West complied more with the guidelines than those living in the South. These results are similar to those from another study, which found that living near the western coast was associated with a higher likelihood of meeting the recommended physical activity levels in England [23]. A possible explanation may be that western coastal areas have more walkable sites, such as parks, beaches, and trails, and milder climates, which could promote year-round physical activity compared with hotter, humid areas that could deter exercise.

### 4.3. Secondary Outcomes

In addition to the socioeconomic and sociodemographic factors included in the analysis, this research included health variables beyond the study’s primary goal to expand knowledge on how different health conditions may affect compliance with recommended physical activity guidelines. The results indicated that those who had chronic obstructive pulmonary disease, emphysema, or chronic bronchitis; those who had some form of arthritis, rheumatoid arthritis, gout, lupus, or fibromyalgia; those with dementia or Alzheimer’s disease; and those with diabetes were less likely to comply with the recommended physical activity guidelines. Previous research agrees that patients diagnosed with diabetes have a lower prevalence of recommended physical activity levels [24]. However, most previous studies have examined physical activity as a therapy for these health conditions rather than these conditions’ impact on adherence to recommended physical activity guidelines, which limits the support for the obtained results. Nonetheless, a possible explanation could be that individuals with chronic conditions such as COPD, emphysema, chronic bronchitis, arthritis, rheumatoid arthritis, gout, lupus, fibromyalgia, dementia, Alzheimer’s, or diabetes often face significant barriers to physical activity compliance [25]. These conditions can cause symptoms such as pain, fatigue, shortness of breath, and reduced mobility, which could make physical activity more challenging, less enjoyable, and even impossible in some cases [26,27,28]. Additionally, these individuals may be at a higher risk of injury or exacerbation of their condition during physical activity, which could lead to fear and avoidance of exercise [29,30,31].

### 4.4. Limitations

Naturally, our study has limitations. Missing data and inaccuracies in the NHIS dataset may affect the validity of the study findings. Furthermore, the NHIS, being a cross-sectional survey, does not permit establishing causal relationships. Additionally, because NHIS relies on self-reported data, the variables used in the models and the reported levels of physical activity may be subject to some degree of misclassification bias. In addition, this study defined compliance with physical activity guidelines as adherence to both strengthening and aerobic guidelines. It did not include separate categories for these, as the number of participants in each category was too small for analysis. Moreover, the survey does not distinguish between type 1 and type 2 diabetes, which are two very different pathophysiological conditions, with type 2 diabetes strongly associated with overweight, obesity, and lack of physical activity. Finally, as participants with missing data on any of the variables were excluded from the analysis, selection bias due to exclusion cannot be ruled out. However, none of the variables included in the analysis had more than 5% of missing data, indicating a very low risk of selection bias due to exclusion.

Despite its limitations, this study has several strengths. First, it focused on a high-interest population that had been understudied and highlighted as important in previous research. Furthermore, this research considered a broad range of socioeconomic and sociodemographic factors, as well as various health conditions that influence physical activity, yielding a comprehensive set of results that can serve as a basis for future research.

### 4.5. Future Directions

Future studies have great potential to deepen our understanding of exercise habits among older adults and to address the challenges they face in maintaining an active lifestyle. One important area of focus could be conducting longitudinal studies. These would be extremely helpful for tracking shifts in physical activity adherence among older adults. They could provide insight into how certain individuals maintain or deviate from recommended physical activity levels over time and help uncover causal relationships. Furthermore, future investigations could benefit from incorporating qualitative research approaches. By conducting interviews, focus groups, and similar methods, researchers can gain a richer understanding of the barriers and facilitators affecting physical activity among older adults. This approach could elucidate personal, social, and environmental factors that either encourage or inhibit physical activity in this age group. Understanding their perspectives can also guide the creation of more supportive interventions. Trans-regional, cross-regional, or cross-cultural comparisons may be another fruitful avenue for research. Researchers could also assess physical activity compliance through the lens of environmental, social, and policy factors across diverse regional, national, and cultural contexts among older adults. Such studies could examine how societal norms, access to exercise facilities, climate, and government policies influence levels of physical activity. Based on these findings, interventions could be tailored to specific contexts, and best practices could be adopted from different cultures or regions. Studying physical activity compliance and barriers is just one focus of future work. Developing and evaluating interventions to enhance physical activity levels among groups with low compliance should also be a focus of future studies. Future research should examine within-group heterogeneity and migration-related factors to better understand structural and contextual drivers of the observed patterns. By combining longitudinal studies, qualitative methods, cross-cultural comparisons, and intervention evaluations, researchers can advance our understanding of how to promote physical activity effectively among older populations. As people age, this knowledge will play a critical role in maintaining their health, independence, and quality of life.

## 5. Conclusions

This study identified several socioeconomic, sociodemographic, and health-related factors associated with adherence to physical activity guidelines among adults aged 65 years and older in the United States. In addition to confirming previously reported associations with sex, age, education, and income, this study highlights novel factors that have received limited attention in previous research: household region, chronic respiratory conditions (COPD, emphysema, or chronic bronchitis), arthritis-related conditions, and dementia or Alzheimer’s disease. These findings suggest that physical activity patterns in older adults are shaped by a complex set of circumstances that extend beyond traditional socioeconomic indicators.

By identifying specific subgroups of older adults who are less likely to meet recommended activity levels—including those with lower income or education, older age, certain chronic conditions, and those living in particular regions—this study provides evidence to inform more tailored interventions. Healthcare providers, public health practitioners, and policymakers can use these results to develop strategies that account for the unique needs, limitations, and environments of these populations. Understanding these patterns is essential for designing equitable, accessible, and effective approaches that support healthy aging in the growing older adult population.

## Figures and Tables

**Table 1:** Characteristics of the sample of adults aged 65 and older according to compliance with the recommended physical activity guidelines for aerobic and strength activities.

Meets Aerobic and Strength Physical Activity Guidelines			
	Does Not Meet Either	Meets Both	*p*-Value
	(n = 6954)	(n = 1235)	
	% (n)	% (n)	
Sex			<0.001
Male	82.6 (2867)	17.4 (629)	
Female	88.1 (4087)	11.9 (606)	
Marital/Partner Status			<0.001
Single	83.6 (3230)	16.4 (684)	
Married/with partner	88.7 (3724)	11.3 (551)	
Age			<0.001
65–74	83.3 (3862)	16.7 (826)	
75–85	89.1 (3092)	10.9 (409)	
Delayed care due to Insurance			0.078
Yes	90.5 (127)	9.5 (19)	
No	85.5 (6827)	14.5 (1216)	
Race			<0.001
Hispanic	90.4 (475)	9.6 (63)	
NH White	84.3 (5458)	15.7 (1044)	
NH Black	89.5 (670)	10.5 (81)	
Other single/multiple races	89.2 (351)	10.8 (47)	
Education level			<0.001
Less than high school	95.7 (790)	4.3 (39)	
High school	92.7 (2097)	7.3 (166)	
Some college/associate degree	86.1 (2009)	13.9 (324)	
Bachelor’s degree	77.1 (1185)	22.9 (334)	
Graduate degree	69.7 (873)	30.3 (372)	
Ratio of family income to poverty threshold			<0.001
Less than 1.00	93.1 (637)	6.9 (53)	
1.00–1.99	92.5 (1584)	7.5 (132)	
2.00–3.99	89.3 (2284)	10.7 (314)	
4.00–4.99	82.7 (721)	17.3 (156)	
5.00 or greater	75.8 (1728)	24.2 (580)	
Time living in a house			0.291
Less than a year	88.3 (304)	11.7 (46)	
1 to 3 years	83.7 (723)	16.3 (140)	
4 to 10 years	85.9 (1306)	14.1 (231)	
more than 10 years	85.7 (4621)	14.3 (818)	
Diabetes			<0.001
Yes	92.5 (1383)	7.48 (125)	
No	83.9 (5571)	16.1 (1110)	
Urban/Rural County			0.003
Large central metro	84.3 (1660)	15.7 (345)	
Large fringe metro	84.2 (1556)	15.8 (304)	
Medium and small metro	85.9 (2388)	14.1 (417)	
Nonmetropolitan	89.2 (1350)	10.8 (169)	
Cancer			0.958
Yes	85.6 (1887)	14.4 (337)	
No	85.6 (5067)	14.4 (898)	
COPD, emphysema, or chronic bronchitis			<0.001
Yes	94.4 (743)	5.6 (55)	
No	84.7 (6211)	15.3 (1180)	
Household region			0.001
Northeast	86.1 (1173)	13.9 (205)	
Midwest	85.2 (1614)	14.8 (293)	
South	87.5 (2597)	12.5 (379)	
West	82.5 (1570)	17.5 (358)	
Arthritis, rheumatoid arthritis, gout, lupus, or fibromyalgia			<0.001
Yes	87.8 (3524)	12.2 (517)	
No	83.6 (3430)	16.4 (718)	
Dementia or Alzheimer’s			<0.001
Yes	96.6 (272)	3.4 (8)	
No	85.2 (6682)	14.8 (1227)	
Heart attack			0.002
Yes	90.0 (573)	10.0 (71)	
No	85.2 (6381)	14.8 (1164)	

**Table 2:** Adjusted and unadjusted association factors with meeting the recommended physical activity guidelines for aerobic and strengthening activity.

Characteristics	Meeting Both Guidelines	
	Unadjusted	Adjusted
	OR (95% CI)	OR (95% CI)
Partner Status		
Married/with partner	Ref.	Ref.
Single	0.6 (0.6–0.7)	1.0 (0.9–1.2)
Age		
65–74	Ref.	Ref.
75–85	0.6 (0.5–0.7)	0.7 (0.6–0.8)
Sex		
Male	1.6 (1.4–1.8)	1.4 (1.2–1.6)
Female	Ref.	Ref.
Race		
Hispanic	0.6 (0.4–0.8)	0.9 (0.7–1.3)
Black NH	0.6 (0.5–0.8)	1.0 (0.7–1.4)
Other single and multiple races	0.7 (0.4–0.9)	0.6 (0.4–1.0)
White NH	Ref.	Ref.
Education		
<High school	0.2 (0.1–0.2)	0.3 (0.2–0.4)
High school	0.3 (0.2–0.3)	0.4 (0.3–0.5)
Some college/associate	0.5 (0.4–0.7)	0.7 (0.6–0.8)
Bachelor	Ref.	Ref.
Graduate	1.5 (1.2–1.8)	1.4 (1.2–1.7)
Ratio of family income to poverty threshold		
<1.00	0.2 (0.2–0.3)	0.6 (0.4–0.9)
1.00–1.99	0.3 (0.2–0.3)	0.6 (0.4–0.7)
2.00–3.99	0.4 (0.3–0.4)	0.6 (0.5–0.8)
4.00–4.99	0.7 (0.5–0.8)	0.9 (0.7–1.1)
5.00 or greater	Ref.	Ref.
Urban/Rural classification for counties		
Large central metro	Ref.	Ref.
Large fringe metro	1.0 (0.8–1.2)	1.0 (0.8–1.2)
Medium and small metro	0.9 (0.7–1.1)	0.9 (0.7–1.1)
Nonmetropolitan	0.7 (0.5–0.8)	0.8 (0.6–1.1)
Household region		
Northeast	1.1. (0.9–1.4)	1.0 (0.8–1.2)
Midwest	1.2. (1.0–1.5)	1.2 (1.0–1.5)
West	1.5 (1.2–1.8)	1.3 (1.1–1.6)
South	Ref.	Ref.
COPD, emphysema, or chronic bronchitis		
Yes	0.3 (0.2–0.5)	0.5 (0.3–0.6)
No	Ref.	Ref.
Arthritis, rheumatoid arthritis, gout, lupus, or fibromyalgia		
Yes	0.7 (0.6–0.8)	0.8 (0.7–1.0)
No	Ref.	Ref.
Dementia or Alzheimer’s		
Yes	0.2 (0.1–0.5)	0.3 (0.1–0.8)
No	Ref.	Ref.
Diabetes		
Yes	0.4 (0.3–0.5)	0.5 (0.4–0.7)
No	Ref.	Ref.
Infarction		
Yes	0.6 (0.5–0.8)	0.8 (0.6–1.1)
No	Ref.	Ref.
Cancer		
Yes	1.0 (0.9–1.2)	-
No	Ref.	-
Delayed medical care due to unaccepted insurance?		
Yes	0.6 (0.4–1.1)	-
No	Ref.	-
Time living in a house		
<1 year	0.8 (0.6–1.1)	-
1–3 years	1.2 (0.9–1.5)	-
4–10 years	1.0 (0.8–1.2)	-
>10 years	Ref.	-

## Data Availability

The original data presented in the study are openly available in NHIS at https://ftp.cdc.gov/pub/Health_Statistics/NCHS/Dataset_Documentation/NHIS/2022/adult-codebook.pdf (accessed on 30 March 2024).

## Abbreviations

The following abbreviations are used in this manuscript:

ADL	Activities of daily living
CIs	Confidence intervals
COPD	Chronic obstructive pulmonary disease
HS	High school
NH	Non-Hispanic
NHIS	National Health Interview Survey
OR	Odds ratio
PA	Physical activity
Ref	Reference group
SES	Socioeconomic status

## Appendix A

**Table A1:** The percentage of missing data for each variable used in the analysis.

Variable	Missing	Total	Percent Missing
outcomepa	354	8771	4.04
sex_a	0	8771	0.00
education	48	8771	0.55
poverty	0	8771	0.00
partner	313	8771	3.57
race	0	8771	0.00
insurance	66	8771	0.75
housing	355	8771	4.05
urbrrl	0	8771	0.00
chronicresp	19	8771	0.22
chronicart	22	8771	0.25
chronicment	14	8771	0.16
diabetes	13	8771	0.15
cancer	19	8771	0.22
infarction	24	8771	0.27

**Table A2:** Variable names, definitions and operational definitions used in this study.

Definition	Variable Name	Variable Definition (Categories/Codes)	Operational Definition for this Study
Age	AGEP_A	18–84 = 18–84 years old, 85 = 85+ years old, 97 = refused, 98 = not ascertained, 99 = do not know	AGEP_A ≥ 65
Met guidelines for aerobic and strengthening activity	PA18_05R_A	1 = meets neither criterion, 2 = meets strength only, 3 = meets aerobic only, 4 = meets both criteria, 8 = not ascertained	4 = meets both criteria 1 = meets neither criterion
Sex	SEX_A	1 = male, 2 = female, 7 = refused, 8 = not ascertained, 9 = do not know	1 = male, 2 = female
Educational level	EDUCP_A	00 = never attended/kindergarten only, 01 = grade 1–11, 02 = 12th grade, no diploma, 03 = GED or equivalent, 04 = high school graduate, 05 = some college, no degree, 06 = associate degree occupational, technical, or vocational program, 07 = associate degree academic program, 08 = bachelor’s degree (BA, AB, BS, BBA), 09 = master’s degree (MA, MS, MEng, MEd, MBA), 10 = professional school or doctorate degree (MD, DDS, DVM, JD, PhD, EdD), 97 = refused, 98 = not ascertained, 99 = do not know	1 ≤ HS, 2 = HS, 3 = some college/associate, 4 = bachelor, 5 = graduate
Ratio of family income to poverty threshold	RATCAT_A	01 = 0.00–0.49, 02 = 0.50–0.74, -3 = 0.75–0.99, 04 = 1.00–1.24, 05 = 1.25–1.49, 06 = 1.50–1.74, 07 = 1.75–1.99, 08 = 2.00–2.49, 09 = 2.50–2.99, 10 = 3.00–3.49, 11 = 3.50–3.99, 12 = 4.00–4.49, 13 = 4.50–4.99, 14 = 5.00 or greater, 98 = not ascertained	1 = less than 1.00, 2 = 1.00–1.99, 3 = 2.00–3.99, 4 = 4.00–4.99, 5 = 5.00 or greater
Partner status	MARITAL_A	1 = married, 2 = living with a partner together as an unmarried couple, 3 = neither, 7 = refused, 8 = not ascertained, 9 = do not know	1 = married/living with a partner as an unmarried couple, 2 = no partner
Race	HISPALLP_A	01 = Hispanic, 02 = non-Hispanic White only, 03 = non-Hispanic Black/African American only, 04 = non-Hispanic Asian only, 05 = non-Hispanic AIAN only, 06 = non-Hispanic AIAN and any other group, 07 = other single and multiple races, 97 = refused, 98 = not ascertained, 99 = do not know	1 = Hispanic, 2 = White NH, 3 = Black NH, 4 = other single/multiple races
Access to healthcare based on insurance	ABINSUR_A	1 = yes, 2 = no, 7 = refused, 8 = not ascertained, 9 = do not know	1 = yes, 2 = no
Housing stability	HOUYRSLIV_A	1 = less than a year, 2 = 1 to 3 years, 3 = 4 to 10 years, 4 = 11 to 20 years, 5 = more than 20 years, 7 = refused, 8 = not ascertained, 9 = do not know	1 = <1 year, 2 = 1–3 years, 3 = 4–10 years, 4 = more than 10 years
Urban–rural classification scheme for counties	URBRRL	1 = Large central metro, 2 = Large fringe metro, 3 = Medium and small metro, 4 = Nonmetropolitan	1 = Large central metro, 2 = Large fringe metro, 3 = Medium and small metro, 4 = nonmetropolitan
Household region	REGION	1 = Northeast, 2 = Midwest, 3 = South, 4 = West	1 = Northeast, 2 = Midwest, 3 = South, 4 = West
Chronic conditions (COPD, emphysema, or chronic bronchitis)	COPDEV_A	1 = yes, 2 = no, 7 = refused, 8 = not ascertained, 9 = do not know	1 = yes, 2 = no
Chronic Conditions (arthritis, rheumatoid arthritis, gout, lupus, or fibromyalgia)	ARTHEV_A	1 = yes, 2 = no, 7 = refused, 8 = not ascertained, 9 = do not know	1 = yes, 2 = no
Chronic conditions (dementia, Alzheimer’s disease)	DEMENEV_A	1 = yes, 2 = no, 7 = refused, 8 = not ascertained, 9 = do not know	1 = yes, 2 = no
Diabetes	DIBEV_A	1 = yes, 2 = no, 7 = refused, 8 = not ascertained, 9 = do not know	1 = yes, 2 = no
Cancer	CANEV_A	1 = yes, 2 = no, 7 = refused, 8 = not ascertained, 9 = do not know	1 = yes, 2 = no
Myocardial infarction	MIEV_A	1 = yes, 2 = no, 7 = refused, 8 = not ascertained, 9 = do not know	1 = yes, 2 = no
